# Signaling through polymerization and degradation: Analysis and simulations of T cell activation mediated by Bcl10

**DOI:** 10.1371/journal.pcbi.1007986

**Published:** 2021-05-20

**Authors:** Leonard Campanello, Maria K. Traver, Hari Shroff, Brian C. Schaefer, Wolfgang Losert

**Affiliations:** 1 Department of Physics, University of Maryland College Park, College Park, Maryland, United States of America; 2 Institute for Physical Science and Technology, University of Maryland College Park, College Park, Maryland, United States of America; 3 Department of Microbiology and Immunology, Uniformed Services University of the Health Sciences, Bethesda, Maryland, United States of America; 4 Laboratory of High-Resolution Optical Imaging, National Institute of Biomedical Imaging and Bioengineering, National Institutes of Health, Bethesda, Maryland, United States of America; National Institutes of Health, UNITED STATES

## Abstract

The adaptive immune system serves as a potent and highly specific defense mechanism against pathogen infection. One component of this system, the effector T cell, facilitates pathogen clearance upon detection of specific antigens by the T cell receptor (TCR). A critical process in effector T cell activation is transmission of signals from the TCR to a key transcriptional regulator, NF-κB. The transmission of this signal involves a highly dynamic process in which helical filaments of Bcl10, a key protein constituent of the TCR signaling cascade, undergo competing processes of polymeric assembly and macroautophagy-dependent degradation. Through computational analysis of three-dimensional, super-resolution optical micrographs, we quantitatively characterize TCR-stimulated Bcl10 filament assembly and length dynamics, and demonstrate that filaments become shorter over time. Additionally, we develop an image-based, bootstrap-like resampling method that demonstrates the preferred association between autophagosomes and both Bcl10-filament ends and punctate-Bcl10 structures, implying that autophagosome-driven macroautophagy is directly responsible for Bcl10 filament shortening. We probe Bcl10 polymerization-depolymerization dynamics with a stochastic Monte-Carlo simulation of nucleation-limited filament assembly and degradation, and we show that high probabilities of filament nucleation in response to TCR engagement could provide the observed robust, homogeneous, and tunable response dynamic. Furthermore, we demonstrate that the speed of filament disassembly preferentially at filament ends provides effective regulatory control. Taken together, these data suggest that Bcl10 filament growth and degradation act as an excitable system that provides a digital response mechanism and the reliable timing critical for T cell activation and regulatory processes.

## Author summary

The immune system serves to protect organisms against pathogen-mediated disease. While a strong immune response is needed to eliminate pathogens in host organisms, immune responses that are too robust or too persistent can trigger autoimmune disorders, cancer, and a variety of additional serious human pathologies. Thus, a careful balance of activating and inhibitory mechanisms is necessary to prevent detrimental health outcomes of immune responses. For example, activated effector T cells marshal the immune response and direct killing of pathogen-infected cells; however, effector T cells that are chronically activated can damage and destroy healthy tissue. Here, we study an important internal activation pathway in effector T cells that involves the growth and counterbalancing disassembly (involving a process called macroautophagy) of filamentous cytoplasmic signaling structures. We utilize image analysis of 3-D super-resolution images and Monte Carlo simulations to study a key signal-transduction protein, Bcl10. We found that the speed of filament disassembly has the greatest effect on the magnitude and duration of the response, implying that pharmaceutical interventions aimed at macroautophagy may have substantial impact on effector T cell function. Given that filamentous structures are utilized in numerous immune signaling pathways, our analysis methods could have broad applicability in the signal transduction field.

## Introduction

The vertebrate adaptive immune system consists of billions of T and B lymphocytes, which serve as a potent and highly specific host defense mechanism against pathogen infection. Once an antigen, an immune-activating component of a pathogen, has been recognized by the T-cell receptor (TCR), an intracellular signaling cascade is initiated that leads to the clonal expansion of T cells responsive to this antigen. This anti-pathogen immune response, including the T-cell component, will cause damage to healthy tissue if not temporally limited [1]. Indeed, unregulated adaptive immune responses have been implicated in the development of certain cancers [1] and autoimmune diseases [2]. Thus, adaptive immune responses include programmed shutdown mechanisms to maintain homeostasis and prevent damage to host tissues [2,3]. In this study, we introduce novel image-analysis tools for the robust analysis of a component signaling pathway of the adaptive immune response from a biophysical perspective, specifically focusing on the spatial organization of a key signaling structure that contributes to adaptive immunity.

The many classes of effector T cells are key regulators of adaptive immunity. Effector T cells are terminally differentiated T lymphocytes with previous antigen exposure that can generate rapid anti-pathogen responses. Antigen engagement of the TCR in effector T cells initiates an internal signal-transduction pathway that leads to the translocation of NF-κB from the cytoplasm to the nucleus. NF-κB is a heterodimeric protein complex that controls gene transcription in response to a diverse array of receptors [4] in both vertebrates and invertebrates [5]. In effector T cells, the nuclear translocation of NF-κB results in the *de novo* or increased expression of a large number of genes involved in T-cell proliferation and immune response mechanisms [5,6]. Improper regulation of NF-κB signaling in T cells is mechanistically associated with primary immunodeficiency [7–9] and autoimmune disease [10,11]. Thus, the regulation of the TCR-to-NF-κB pathway is critically important for proper immune function.

A key signaling protein in the TCR-to-NF-κB pathway is Bcl10 [12]. Upon TCR stimulation, Bcl10 assembles with its signaling partners Carma1 and Malt1 to form the micron-scale ‘CBM’ complex [13–17]. In effector T cells, the CBM complex forms the core of a filamentous assembly called the POLKADOTS signalosome, which serves as the cytoplasmic site of the terminal steps of the NF-κB-activation cascade [13,14,18–21]. At the same time that Bcl10 assembles into microns-long filaments, it is also being degraded [13,22–26]. We have previously shown that proteolysis of Bcl10 occurs within the POLKADOTS signalosome via TCR-dependent selective autophagy [18]. In this degradative process, autophagosomes associate with POLKADOTS filaments, resulting in selective destruction of Bcl10, and thus terminating signals to NF-κB. Data suggest that the balance between assembly of filamentous Bcl10 in POLKADOTS and proteolytic degradation of Bcl10 via selective autophagy determines the extent of NF-κB activation [13,18].

Notably, many outputs of TCR signaling exhibit nonlinear response profiles, including an all-or-nothing (i.e., digital) response. Although not every T cell response has digital characteristics [27], studies have demonstrated that specific TCR-triggered events are digital, including activation of the extracellular regulated kinase-mitogen activated protein kinase (ERK-MAPK) signaling cascade [28–30], signaling via the protein kinase D2-protein kinase C (PKD2-PKC) cascade [31], release of cytokines [32], and activation of cytolytic capacity [33]. Additionally, we have previously shown that TCR activation of NF-κB is digital in nature, with formation of and signal transmission by the POLKADOTS signalosome occurring in an all-or-nothing manner [34]. Mechanistically, how nucleation, growth and eventual autophagic degradation of POLKADOTS filaments are connected to the digital nature of the NF-κB cascade is unclear. In this manuscript, we link the nonlinear character of the signaling cascade to the spatial organization, assembly, and degradation of filamentous Bcl10. We propose that highly nonlinear self-assembly and degradation processes comprise an excitable system, enabling a rapid yet self-limiting, response.

Several key details of this polymerization process are known: recent cell-free cryo-electron-microscopy studies revealed that Bcl10 polymerizes into filaments in a geometrically confined, helical arrangement with other POLKADOTS components [14,20,21]. Although the core of the POLKADOTS filament is composed primarily of Bcl10, polymerization of Bcl10 is nucleated by short oligomers of Carma1, and the Carma1-driven nucleation of Bcl10 can act as an amplifier of activation signals from the T-cell receptor to NF-κB [14]. In contrast to the above parameters driving filament growth and signal propagation, autophagic degradation of Bcl10 opposes this amplification process. However, the geometric and kinetic features that limit, and ultimately reverse, filament growth in living T cells are unclear. These opposing effects on filament growth and degradation are not well understood and cannot be assessed via traditional biochemical approaches and signaling pathway models, which exclude spatial organization.

In this study, we quantify the spatial arrangement of Bcl10, its colocalization with autophagosomes, and the randomness of their relative spatial distributions. Our analysis introduces a novel computational workflow that combines bootstrap-like resampling methods adapted for image features and medial-axis-thinning skeletonization for the robust analysis of the structure and organization of Bcl10 filaments in relation to autophagosomes. To gain further mechanistic insights into the assembly and degradation dynamics of POLKADOTS filaments, we complement analysis of super-resolution images with stochastic Monte Carlo simulations of POLKADOTS dynamics that include the nucleation, growth, and disassembly of filamentous Bcl10. In sum, we show that the spatial assembly and disassembly of protein complexes in the TCR-to-NF-κB-activation cascade are key design elements to ensure a rapid response yet prevent ongoing immune activation. These elements may also contribute to the digital nature of this crucial signaling pathway. The methods we present are broadly applicable to studies of biologically encoded spatial colocalizations and self-assembly, such as toll-like-receptor-stimulated assembly of the myddosome, RIG-I-like-receptor triggering of mitochondrial-antiviral-signaling-protein oligomerization, and activation of the various sensor proteins that promote assembly of micron-scale inflammasomes [35].

## Results

### Measuring Bcl10 filament lengths

**Fig 1:**
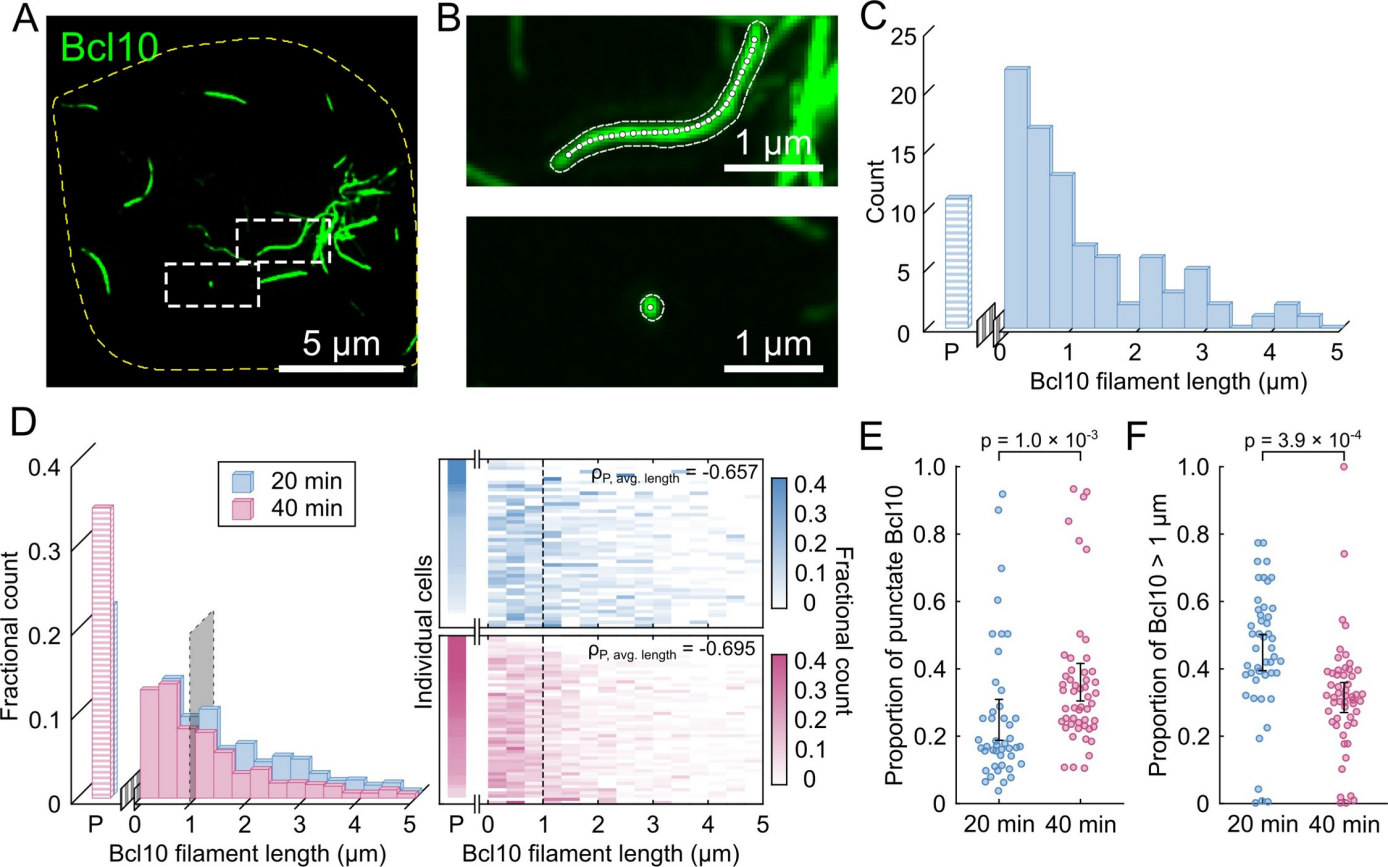
Bcl10 filaments shorten between 20 min and 40 min post-T cell receptor activation. (A) Representative image (maximum intensity projection) of an activated T cell 20 min after TCR stimulation. The outlined region of interest contains two complete Bcl10 filaments. (B) Representative Bcl10 filaments within the activated T cell. Filament outlines and skeletons were calculated using a semiautomatic segmentation method and medial axis thinning. The upper image exemplifies the filamentous form of Bcl10 and the lower image is representative of punctate Bcl10. (C) Representative distribution of Bcl10 lengths from all Bcl10 filaments from (A). Punctate Bcl10 structures were separately counted and binned as “P”. (D) Cumulative length distributions of all Bcl10 filaments in all T cells at 20 min (N = 47 cells from 2 independent experiments) after TCR stimulation and 40 min (N = 53 from 2 independent experiments). Each row of the heat map is the length distribution for a cell sorted by the percentage of punctate-Bcl10 structures in each cell. The correlations (ρ) between the number of punctate structures and average filament length are -0.657 and -0.695 at 20 min and 40 min post-activation, respectively. (E) Scatter plot of the proportion of Bcl10 structures which are punctate at 20 min and 40 min after TCR stimulation. (F) Scatter plot of the proportion of Bcl10 filaments longer than 1 μm at 20 min and 40 min after TCR stimulation. Correlations are the Pearson product-moment correlation. Error bars represent the 95% confidence interval of the mean. *p*-values were calculated using a two-sample *t*-test. *p*-values less than 0.05 were considered significant.

Utilizing a long-term murine effector T cell clone, D10.G4.1 (henceforth referred to as D10), we engineered a cell line stably expressing GFP-tagged Bcl10, which we used to image Bcl10 filaments in cells fixed at 20- and 40-min post-TCR activation using a super-resolution instant structured illumination microscope (iSIM) [36] (**Fig 1A**). The earliest evidence of formation of POLKADOTS structures is at 10 min post-TCR activation. These polymers are maximally evident by 20 min post-activation, at which point initial interaction with autophagosomes is also apparent. Association with autophagosomes and degradation of Bcl10 persists through 2 hr post-stimulation, by which time Bcl10-containing polymers can no longer be found in any cells. Indeed, these polymers are rare even by 60 min post-activation [13,15]. Thus, 40 min post-activation is a reasonable time point at which to assess intermediate consequences of autophagosome association with Bcl10 filaments.

To analyze these data, we developed a semiautomatic segmentation and skeletonization algorithm based on medial-axis thinning. We used the resultant skeletons to make various structural measurements (**Methods** and **S1 Movie**). Bcl10-rich regions with skeleton lengths <150 nm (the approximate spatial resolution of the iSIM) were designated as punctate structures, labeled by “P” in these data. The punctate structures were analyzed separately from micron-scale filaments (**Fig 1B**), the latter of which ranged in lengths up to 5 μm (**Fig 1C**). Between 20 min and 40 min post-activation, the relative number of Bcl10 puncta increased, and the number of long Bcl10 filaments correspondingly decreased (**Fig 1D**). Furthermore, at both time points, a moderate negative correlation (ρ ≈ -0.7) existed between the overall number of Bcl10 puncta and the distribution of Bcl10 filament length. Thus, cells with fewer puncta were more likely to have long filaments, and cells with more puncta were more likely to have shorter filaments (**Fig 1D**). This correlation existed and was statistically significant at the single-cell level. Cells at 40 min post-stimulation were more likely to have both a larger relative proportion of punctate structures (**Fig 1E** and **S1 Table**, p = 1.0 × 10^−3^), and a decrease in the relative number of Bcl10 filaments longer than 1 μm (**Fig 1F** and **S1 Table**, p = 3.9 × 10^−4^). These results are consistent with the interpretation that between 20 min and 40 min post-activation, Bcl10 filaments decrease in length and the proportion of punctate structures increases. This increase in the number of punctate structures and decrease in the number of long filaments could indicate: (i) ongoing nucleation of new filaments; (ii) disassembly or end-directed degradation of existing filaments; (iii) scission of existing filaments, which would create two shorter “daughter” filaments with each scission event; or (iv) some combination of the above processes.

### Examining autophagosome-Bcl10-filament interactions

**Fig 2:**
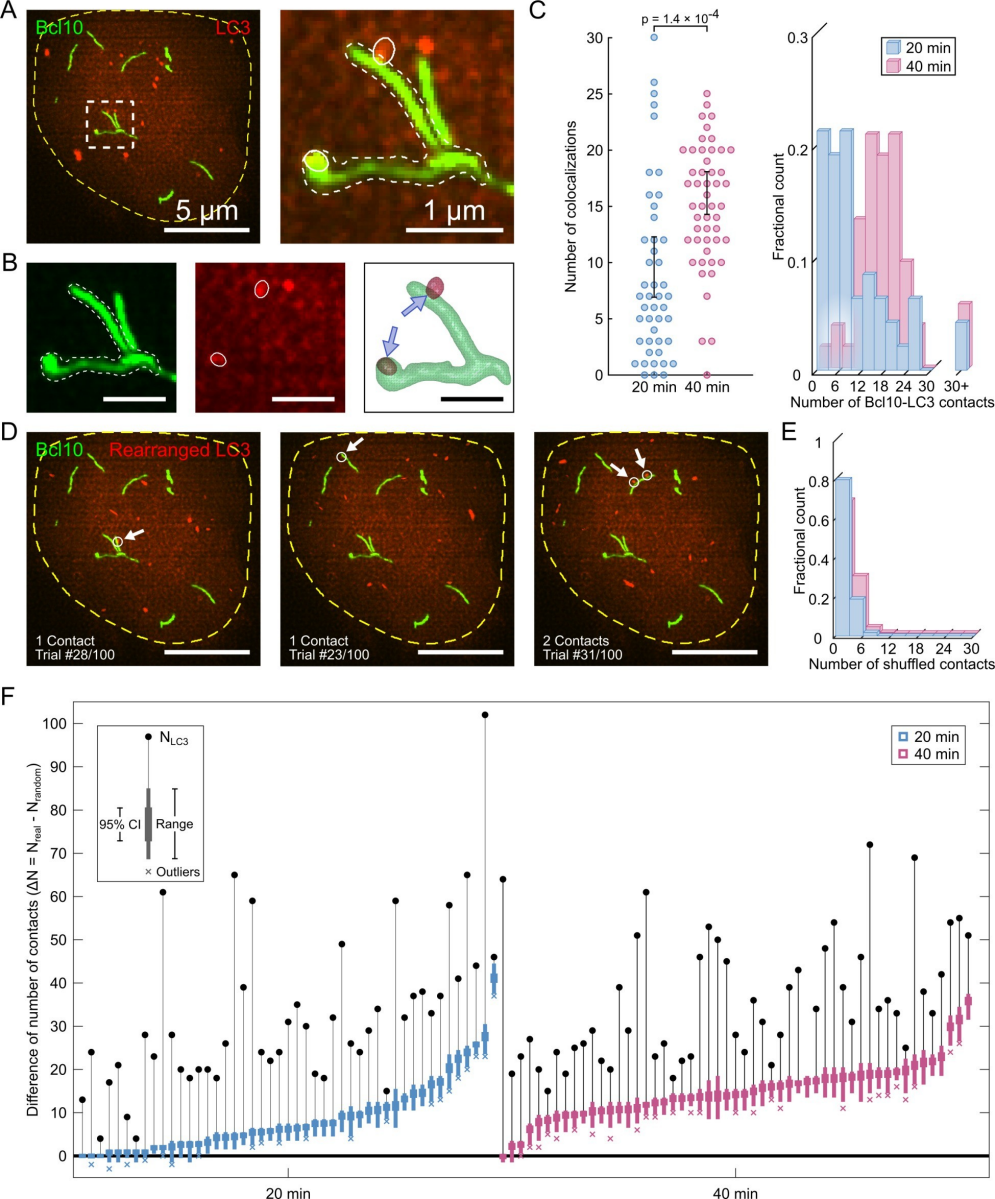
Bcl10 filaments are preferentially in contact with autophagosomes. (A) Representative image of an activated T cell 20 min after TCR stimulation. Green Bcl10 filaments and red LC3 vesicles (autophagosomes) are within the yellow cell boundary. The outlined region of interest contains a Bcl10 structure with two colocalized autophagosomes. (B) Representative Bcl10 filament-autophagosome contacts. The semiautomatic segmentation and skeletonization analyses identify the number and location of colocalizations which are highlighted by blue arrows. (C) Cumulative number of Bcl10 filament-autophagosome contacts in all T cells (20 min: N = 47 cells from 2 independent experiments; 40 min: N = 53 cells from 2 independent experiments). There is a statistically significant difference in the number of Bcl10-autophagosome contacts at 20 min and 40 min post-stimulation (p = 1.4 × 10^−4^). Error bars are 95% confidence intervals of the mean. (D) Three representative trials of the random-spatial rearrangement of autophagosomes in the representative cell in (A). Contacts that formed after rearrangements are highlighted with arrows. (E) The cumulative distribution of the number of Bcl10 filament-autophagosome contacts in the random rearrangement trials. (F) Difference between the true number of Bcl10 filament-autophagosome contacts and the number of contacts found in each of the 100 trials for each cell. Positive values indicate a greater number of true contacts than found in the trials. There is a statistically significant difference in the number of contacts between the true and rearranged data in 45 of 47 T cells imaged 20 min post-activation, and 52 of 53 T cells imaged 40 min post-activation. Error bars are the 95% confidence interval of the mean and were calculated independently for each cell. *p*-values were calculated using two-tailed *t*-tests. *p*-values less than 0.05 were considered significant. Scale bars in (A–left) and (D) are 5 μm, and in (A–right) and (B) are 1 μm.

Our previous work has demonstrated that Bcl10 within the filamentous POLKADOTS signalosome is targeted for degradation by macroautophagy (henceforth referred to as autophagy) [18], an intracellular degradative process involving the envelopment of cargo by double-membraned vesicles called autophagosomes. Autophagic degradation of Bcl10 leads to a dampening of NF-κB activation and NF-κB-dependent T-cell responses [18]. To examine whether autophagy is responsible for the observed decrease in Bcl10 filament length, we expressed an RFP-tagged form of the autophagosome membrane protein LC3 in our Bcl10-GFP-expressing D10 cell line, then imaged static interactions between Bcl10 filaments and LC3-labeled autophagosomes at 20 min and 40 min post-TCR activation (**Fig 2A**). Consistent with our previous data [18], these experiments indicated that filamentous Bcl10 and LC3-positive autophagosomes existed simultaneously in activated T cells. A large number of autophagosome contacts with Bcl10 filaments were observed. Thus, independent semiautomatic segmentation of Bcl10 filaments and autophagosomes was used to determine the number and location of contacts (**Fig 2B**). Interestingly, we observed that activated T cells have significantly fewer Bcl10-autophagosome contacts at 20 min post-TCR stimulation than at 40 min post-stimulation (**Fig 2C** and **S2 Table**, p = 1.45 × 10^−4^). However, this fact alone is not sufficient to conclude that contact formation is non-random. To assess whether autophagosome contacts with Bcl10 filaments form preferentially or randomly, we developed an image-based-resampling method analogous to statistical bootstrapping (**Methods**). We randomly rearranged the segmented autophagosomes throughout the cell cytosol and recalculated the resulting filament-autophagosome contacts (**Fig 2D**). The cell-averaged number of colocalizations in these rearrangements was significantly fewer than the number in the experimental observations (**Fig 2E**). As an additional test, we conducted 100 rearrangements of each individual cell and constructed a confidence interval for the number of randomized contacts. The actual number of autophagosome contacts in 45 of 47 cells at 20 min post-activation, and 52 of 53 cells at 40 min post-activation, was greater than the 95% confidence interval for random LC3 contacts (**Fig 2F**). Thus, we concluded that contact between Bcl10 and autophagosomes was non-random, and that there were indeed more Bcl10-autophagosome interactions at 40 min post-activation.

Our results thus far indicate that at 40 min post-activation, Bcl10 filaments are both shorter and have increased frequency of contact with autophagosomes, consistent with the interpretation that autophagosomes degrade the filaments over time. However, a mechanistic problem arises from these data: autophagy occurs when double-membraned vesicles completely surround and envelop intracellular cargo, yet Bcl10 structures are larger than autophagosomes–the majority of autophagosome structures observed in these cells are smaller than (0.2 μm)^3^ in volume at both 20 min and 40 min post-activation (**S1 Fig**). Thus, the traditional biophysical understanding of mechanisms of autophagy may not be viable as an explanation for Bcl10-filament degradation.

**Fig 3:**
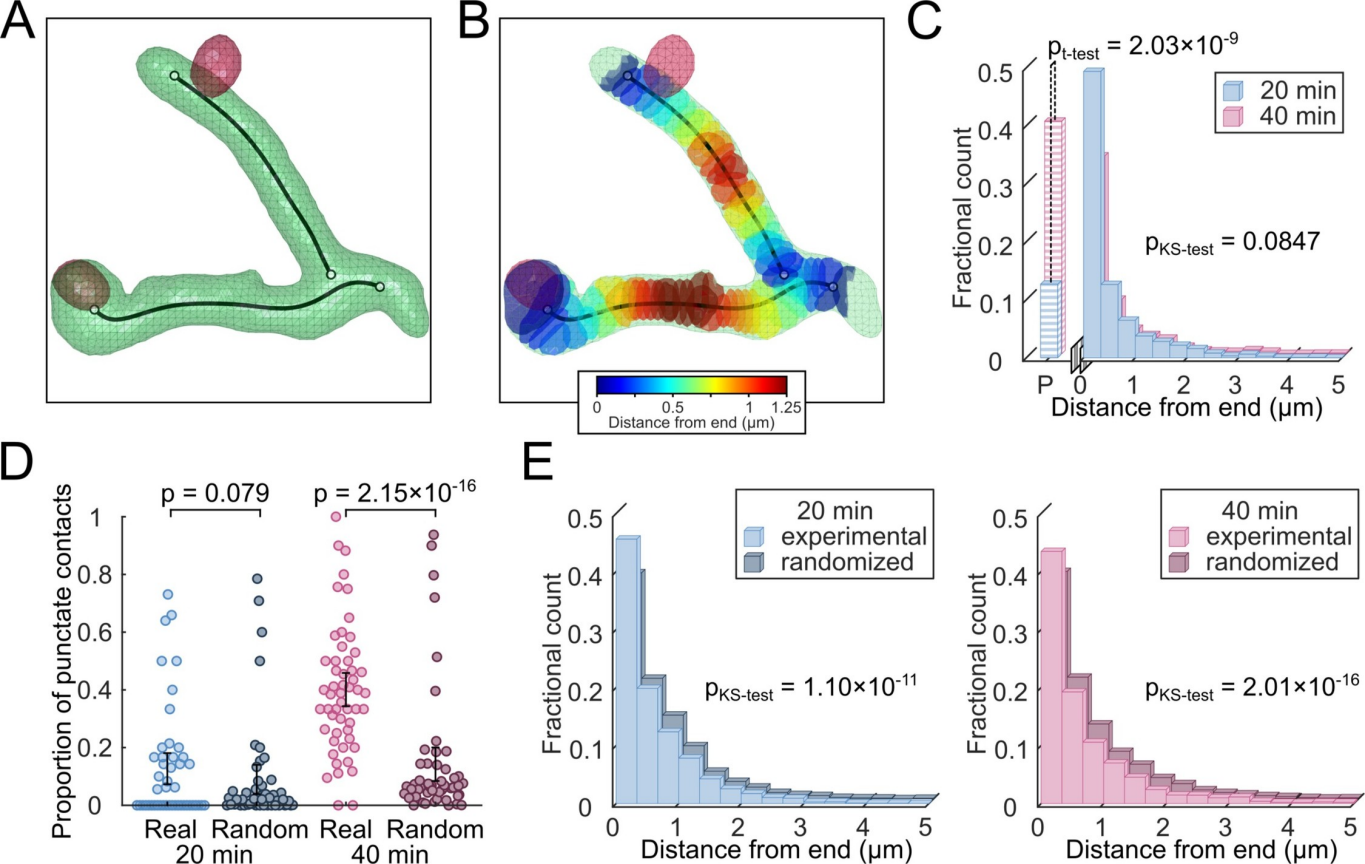
Autophagosomes preferentially localize to Bcl10-filament ends and, at later timepoints, to Bcl10 puncta. (A) Representative segmented Bcl10 filaments (green) and autophagosomes (red) after post-processing, overlaid with the pruned medial-axis skeleton of Bcl10 (black). Filament skeletons were fragmented to minimize the bending energy at junctions. (B) Using the skeleton of the representative Bcl10 filament, the filament volume was segmented into sections based on the nearest distance from skeleton ends. (C) Distribution of autophagosome-Bcl10 contacts by distance from endpoint (filamentous Bcl10) or punctate Bcl10 (“P”). (D) Scatter-bar plot of the proportion of Bcl10 puncta per cell in contact with an autophagosome in experimental and randomized data (i.e., puncta only). (E) Rescaled distance-from-endpoint distributions from (C) of only autophagosome-filamentous Bcl10 colocalizations in experimental and randomized data (i.e., puncta removed). *p*-values in C and E were calculated using two-sample KS tests and *p*-values in D were calculated using paired two-sample *t*-tests. *p*-values less than 0.05 were considered significant.

To examine the mechanism whereby autophagosomes degrade Bcl10 filaments, we examined the location of autophagosome colocalizations along each filament. Biochemical and structural studies have demonstrated that Bcl10 filaments have two distinct end points and no junctions along the main body [14]. However, due to the resolution limits of this imaging method, some filaments appear joined. Thus, employing the same super-resolution dataset used for **Fig 2**, we extracted Bcl10 filament skeletons and fragmented them to eliminate high-degree junctions such that the resulting skeleton configurations minimized the total bending energy (**Fig 3A** and **Methods**). After processing the skeletons, each Bcl10 filament was segmented based on the distance from the nearest skeleton endpoint (**Fig 3B**). Finally, we examined each colocalization event between an autophagosome and a Bcl10 structure. If the Bcl10 structure was punctate, the event was designated “P”, and if the Bcl10 structure was filamentous, the event was characterized by the shortest distance between the autophagosome and the skeleton endpoint (**Fig 3C**). Excluding puncta, the distance-from-end distributions at 20 min and 40 min were not statistically distinguishable (**Fig 3C** and**S3 Table**, p = 0.085), and we found a marked preference for autophagosomes to localize near filament ends at both time points. Interestingly, a greater proportion of autophagosomes were in contact with Bcl10 puncta (as opposed to filaments) at 40 min post-activation than at 20 min post-activation (**Fig 3C** and **S3 Table**, p = 2.03 × 10^−9^). Although the proportion of punctate Bcl10 approximately doubled between 20 and 40 min post-activation (**Fig 1E**), the proportion of autophagosomes that localized to puncta approximately quadrupled over the same period (**Fig 3C**), indicating a distinct preference for autophagosomes to localize with punctate Bcl10 at the later time point.

Our observations thus far indicate that autophagosomes are more likely to localize to the ends of filaments and, by 40 min post-activation, to Bcl10 puncta. To assess whether these localizations are non-random, we again employed the randomized images from the image-based resampling shown in **Fig 2D**. First, we compared the number of colocalizations of Bcl10 puncta with autophagosomes in the true versus the randomized images (**Fig 3D**). At 20 min post-activation, the true and random distributions of contacts were indistinguishable (**Fig 3D** and **S3 Table**, p = 0.079), indicating that autophagosome-puncta contacts could be the result of random co-localization. However, at 40 min post-activation, the number of true puncta contacts was greater than the number of such contacts in the randomized images, and this enrichment was statistically significant (**Fig 3D** and **S3 Table**, p = 2.15 × 10^−16^), indicating that punctate Bcl10 structures were preferentially in contact with autophagosomes at this time point. Next, we compared the distance-from-end distributions of autophagosome-filament contacts for the experimental versus randomized data at both time points (**Fig 3E**). The randomized distributions at both time points demonstrated fewer end-localizations and increased numbers of contacts along the body of the filament in comparison to quantification of localization data from true images (**Fig 3E** and **S3 Table**, p = 1.10 × 10^−11^ and 2.01 × 10^−16^ at 20 min and 40 min, respectively). These results indicate that the preference for autophagosomes to localize at or near filament ends is non-random.

Taken together, the results from **Figs 2** and **3** indicate that following TCR engagement and activation, autophagosomes formed attachments with Bcl10 filaments, the number of attachments increased over time, and these attachments occurred near the ends of Bcl10 filaments. Furthermore, by 40 min post-activation, autophagosomes also formed attachments with Bcl10 punctate structures in a biologically targeted (i.e., non-random) manner. Together, these results are consistent with the hypothesis that autophagosomes are responsible for the progressive shortening of Bcl10 filaments over time, and that this process is directed by a biologically encoded targeting process. Further, the accumulation of punctate Bcl10 structures at 40 min post-activation may be the result of the degradation of longer Bcl10 filaments. Complete degradation of Bcl10 may follow nonlinear kinetics, with the final remnant, visualized as puncta, undergoing slower degradation than filamentous regions of these structures.

### Modeling Bcl10 filament dynamics

Our previously published observations demonstrate that macroautophagy contributes to Bcl10 filament degradation [18], and the above analyses of imaging data supports and extends these conclusions. This degradation occurs simultaneously with Bcl10 polymerization, and the interplay between these processes serves to control T-cell activation states. However, our understanding of the dynamics of filament polymerization and degradation, and the interaction between these processes, remains limited.

**Fig 4:**
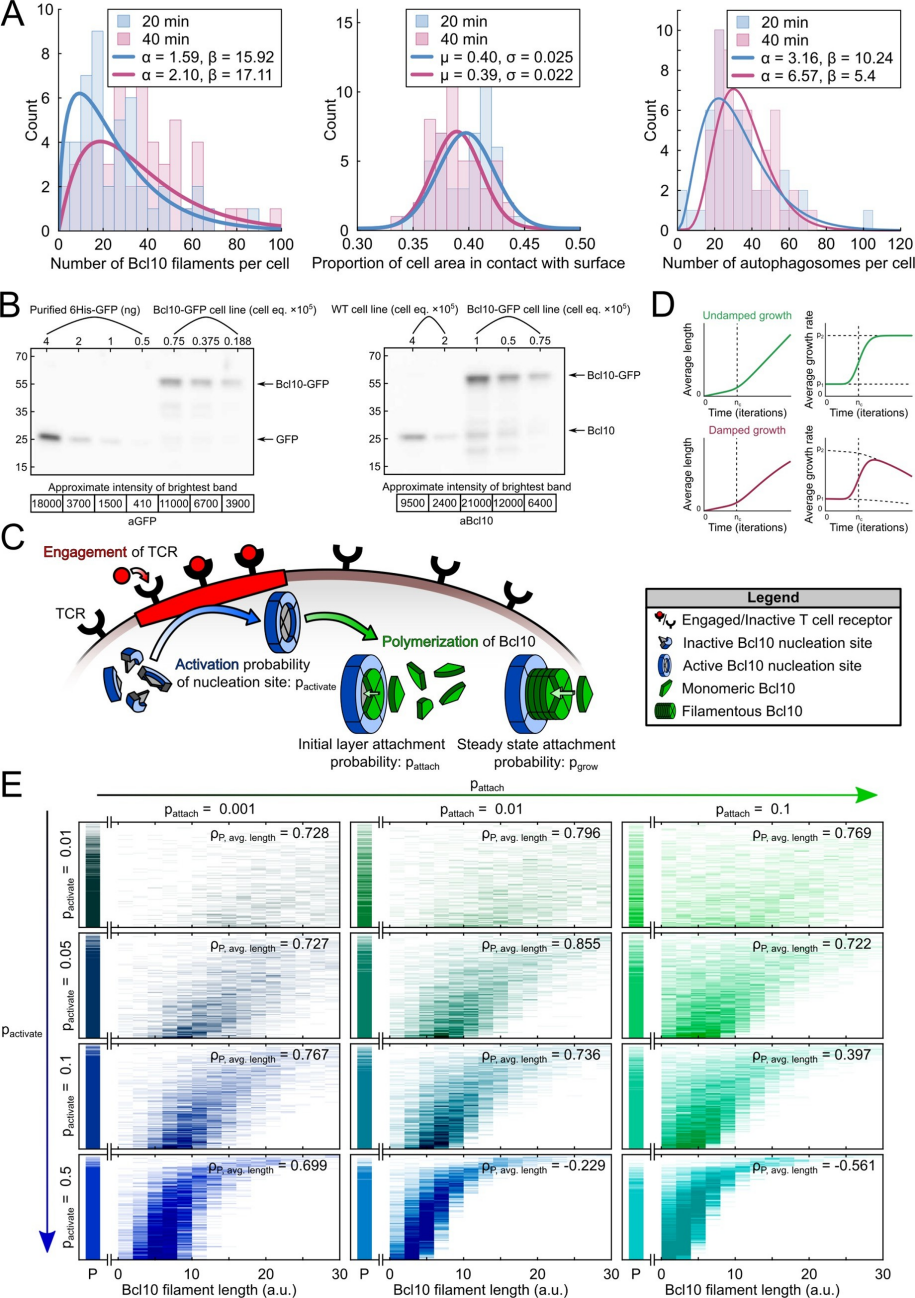
Simulations of Bcl10 filament growth reveal properties of real Bcl10 nucleation rates and concentrations, but fail to match observed values for ρ. (A) Measurements of 20- and 40-min image data used to generate simulated cells with varying initial conditions. Simulated cells were initialized with a random number of Bcl10 nucleation sites that was sampled from a gamma distribution with parameters 1.59 and 15.92 (from the 20-min data); the randomly generated size of the immunological synapse relative to the size of the cell was sampled from a normal distribution with mean 0.40 and standard deviation 0.025; and the number of autophagosomes in the cell was sampled from a gamma distribution with parameters 4.3 and 7.9, which is result of combining the gamma distributions that best-fit the 20- and 40-min data. (B–left) The indicated amounts of purified histidine-tagged GFP or number of cells were processed for western blotting with an anti-GFP antibody. Approximate intensity of the highest intensity band is shown. Amount of GFP vs. band intensity was used to generate a standard curve, from which the amount of GFP in the given number of Bcl10-GFP-expressing cells was interpolated. (B–right) The indicated number of wild-type or Bcl10-GFP-expressing cells was processed for western blotting with an anti-Bcl10 antibody. Approximate intensity of the highest intensity band is shown. These values were used to determine the approximate fold-increase in Bcl10 expression in the exogenously expressing cell line. (C) Simulated Bcl10-filament growth is controlled by three parameters: the nucleation-site activation probability (p_activate_), the initial layer attachment probability (p_attach_), and the steady state attachment probability (p_grow_). (D) Average filament length and associated filament growth rates in a two-state nucleation-limited growth scenario. The undamped growth case (green, top) is when monomer access is not the rate-limiting step for filament growth, and the damped case (red, bottom) is when monomer access damps continued growth. (E) Changing simulation parameters exposes different behaviors in a heterogeneous population of T cells. Here, the nucleation-site-activation and initial-layer-attachment probabilities are simultaneously modulated in the same heterogenous population of 100 T cells with varying numbers of nucleation sites and initial Bcl10 concentrations. From left to right, the Bcl10 collision rate is increased. From top to bottom, the activation rate of nucleation sites is increased. All simulations are terminated when the concentration of Bcl10 monomers reaches zero.

To explore the interplay between simultaneous Bcl10 polymerization and degradation further, as well as the implications of this interplay for control of T-cell-activation states, we designed a stochastic Monte Carlo simulation to model Bcl10 polymerization (**Fig 4** and **Methods**). To create our simulation, we first utilized our extensive imaging data to determine the range of initial conditions from which the simulation would begin, including the number of filaments per cell (used to determine the number of nucleation sites), the proportion of the cell surface area in contact with the activating surface (used the determine the size of the immunological synapse), and the number of autophagosomes per cell (**Fig 4A**). Additionally, we determined the approximate number of Bcl10 molecules per cell prior to activation via quantitative western blotting (**Fig 4B**). Our simulation began with the generation of a “cell,” whose starting parameters were governed by the distributions outlined in **Fig 4A** and **4B**. As this cell evolved in time over each iteration of the simulation, the growth of Bcl10 filaments was governed by three independent and tunable parameters (visually represented in **Fig 4C**): the activation probability of the nucleation sites (p_activate_), the growth probability of the initial layer of Bcl10 monomers before the nucleation barrier has been overcome (p_attach_), and the steady-state growth probability after the filament overcomes the nucleation barrier (p_grow_).

Previous studies have indicated that growth of Bcl10 filaments is nucleated by short cytosolic oligomers of Carma1 [14,20], and that this nucleation is the rate-limiting step for filament growth [14]. Thus, we used nucleation-limited filament assembly as the basis for our simulation, building on successful prior studies of microtubules [37]. Nucleation-limited assembly is similar to a Bernoulli process whose probability parameter increases when the length of the growing filament reaches a critical length: the size of the nucleation barrier. If we define *n* (the number of attempted attachments, i.e., iterations) and L_0_ (the size of the nucleation barrier), then the probability of a filament having length *x* due to simple nucleation-limited growth will have the following distribution:

$$(n)=\{(n\;x)p_{attach}^{x}{\left(1-p_{attach}\right)}^{n-x},x<L_{0}\sum_{i=L_{0}}^{n-(x-L_{0})}(i-1L_{0}-1)p_{attach}^{L_{0}}{\left(1-p_{attach}\right)}^{i-L_{0}}(n-i\;x-L_{0})p_{grow}^{x-L_{0}}{\left(1-p_{grow}\right)}^{\left(n-i)-(x-L_{0}\right)},x\geq L_{0}.$$ (1)

These theoretical predictions indicate a dramatic transition at a critical point, *n*_*c*_, between slow, initial growth, to fast, steady-state growth (**Fig 4D**). Furthermore, since filament growth is mediated by a kinetic process whereby monomers collide with (or are recruited to) the site of the growing filament, as the concentration of free Bcl10 monomers decreases over time then the probability of monomer collisions with the growing filament end also decreases over time (**Fig 4D–bottom**). Thus, as access to monomeric Bcl10 decreases due to filament growth, the growth rate will slowly decrease.

The next step in the development of our simulation was to determine whether changes in the value of p_grow_ would provide useful information regarding the response dynamics. Previous work by David et al. [14] indicated that nucleation (p_activate_) and/or the initial attachment of Bcl10 monomers (p_attach_) is the rate-limiting step in filament growth. Thus, varying the value of p_grow_ would likely provide little in the way of relevant biological information. Furthermore, we found that increasing the growth rate rapidly depleted the monomer concentration, thus ending the simulation after fewer iterations and limiting the sampling of the activation and attachment probabilities. Thus, we chose to assign p_grow_ a fixed value that allowed the simulations to run for a reasonable number of iterations (here, we chose p = 0.4 as described in **Methods**), and we only modulated p_activate_ and p_attach_.

Next, we allowed our simulation to run to completion using 100 initial parameter sets, while varying the values for p_activate_ and p_attach_ in a systematic fashion to determine their respective effects on the resultant filament length distribution (**Fig 4E**). The observed filament lengths in our cell images (**Fig 1D)** demonstrated moderately negative correlations between the relative numbers of puncta and length of Bcl10 filaments, ρ ≈ -0.7. We therefore explored which combinations of parameters applied to the same heterogenous population of 100 simulated T cells could generate an equivalent correlation. Each simulation was repeated 10 times, and each trial was terminated when the number of free monomers of Bcl10 reached zero. The filament-length distributions we report represent the cumulative distribution from each of the 10 trials. Our results indicate that p_activate_ has a far greater effect on filament length than p_attach_ (Fig 4E and S4 Table). As p_activate_ increases, filament lengths decrease, a result that makes intuitive sense as an increased number of filament growth sites would result in the limited pool of Bcl10 monomers being divided between more filaments (Table A in **S4 Table**). Increasing p_attach_ similarly led to a decrease in filament size, but the effect was much less pronounced (Table B in **S4 Table**). In contrast to the generally large positive values for ρ under most simulation conditions, when the probabilities of both activation and attachment were high (0.5 and 0.1, respectively), the correlation between puncta number and filament length was -0.561, which approached but failed to reach the experimentally observed levels of ρ in **Fig 1D**; furthermore, the vast majority of these simulated cells did not reflect the filament length heterogeneity found in our cell images (**Fig 1D**), instead containing large numbers of short filaments (**S4 Table**). This failure to match the model outcomes to experimental observations points to a missing, necessary factor in the simulations: Bcl10 filament disassembly, involving degradation via macroautophagy.

**Fig 5:**
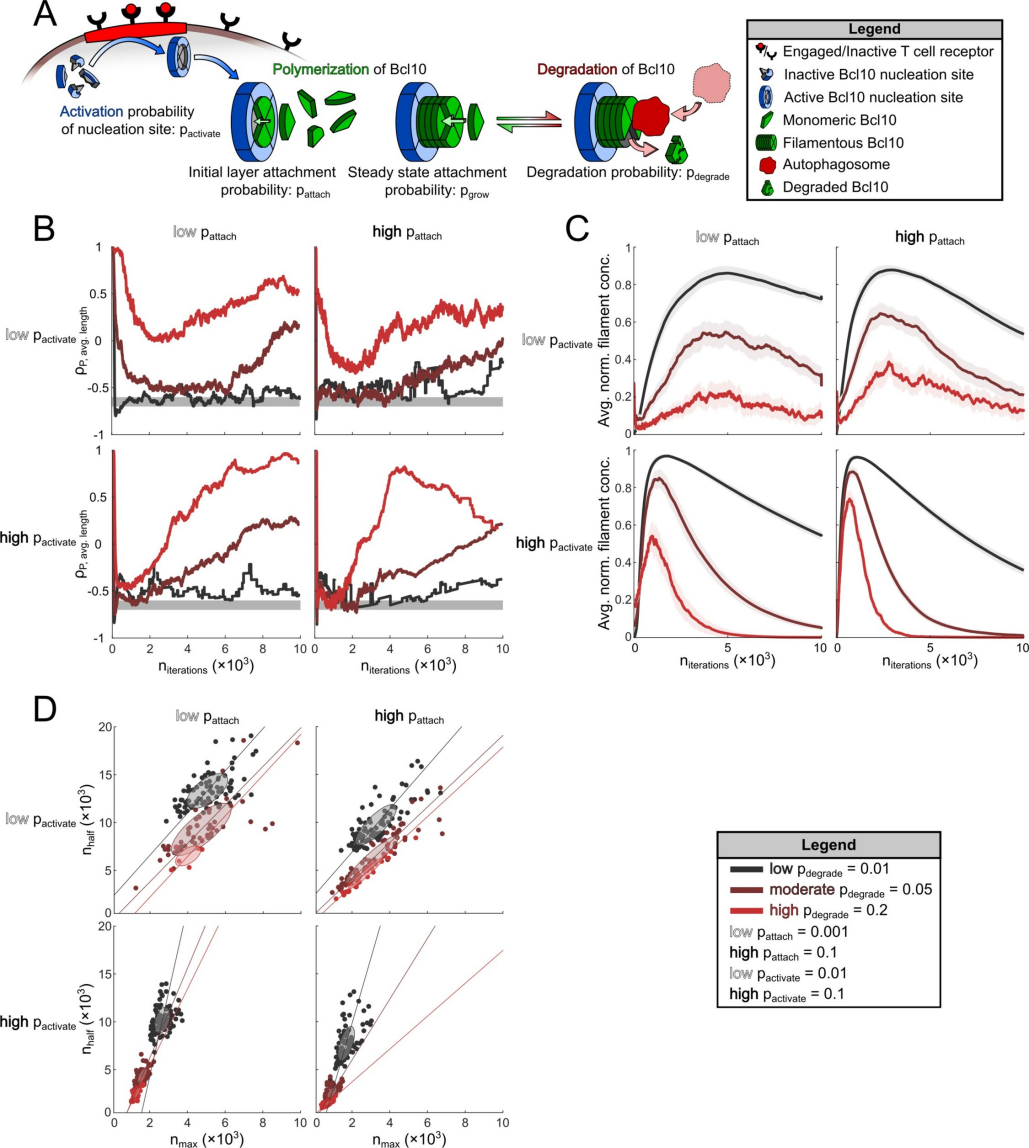
Simulations of Bcl10-filament growth and decay demonstrate that changes in the response dynamic due to increased degradation by autophagy were stabilized by increased p_attach_ but emphasized by increased p_activate_. (A) Bcl10-filament degradation was simulated in conjunction with filament growth. Degradation was modulated by p_degrade_, the probability of degradation by an autophagosome. (B) The over-time correlation (ρ) between the relative concentration of punctate Bcl10 and the average length of Bcl10 filaments for low, moderate, and high values of p_degrade_. The gray band spans from ρ = [-0.7, -0.6], which is the approximate range of the experimentally observed ρ at both 20- and 40-min post activation. Each line represents the accumulation of data for 100 cells, repeated 10 times each. Simulations were repeated with low and high values for p_activate_ and p_attach_. (C) The average normalized concentration of filamentous Bcl10 as simulations evolved. Concentration is relative to the maximum-average concentration of all simulations with the same parameters. Shaded regions are the 95% CI of the mean. (C) The number of iterations needed to reach the maximum relative concentration (n_max_) vs. the number of iterations to reach half of the maximum concentration (n_half_) due to degradation. Each entry represents the average n_max_ and n_half_ for the 10 trials of each of the 100 cells. In (B), (C), and (D), Low p_degrade_ is 0.05, moderate p_degrade_ is 0.10, and high p_degrade_ is 0.20. Ellipses in (D) represent one standard error of the mean.

To understand the effect of degradation on Bcl10 filament size, we modified the Monte Carlo simulation by adding autophagosome-led degradation. The per cell number of autophagosomes was based on the number of autophagosomes observed in real cells via super-resolution imaging (**Fig 4A**). Based on our extensive observations of autophagosome-filament interactions in both fixed and live cells, we designed the simulation to reflect multiple possible outcomes of autophagosome-filament interaction (see Methods for full details). In brief, autophagosomes that came in contact with a filament could lead to removal of Bcl10 monomers from the growing end, or they could cause scission of the filament into two daughter filaments. The probability that an autophagosome would remove a monomer or cut the filament was represented by a new simulation parameter, p_degrade_ (**Fig 5A**).

We first examined whether this more advanced simulation would generate correlation values of filament length vs number of puncta that more closely matched our observational data (**Fig 1D**). We chose a low vs a high value for both p_attach_ (**Fig 5B, left vs right**) and p_activate_ (**Fig 5B, top vs bottom**). For each of these four parameter subspaces, we then compared a low, medium, and high value for p_degrade._ Several parameter configurations led to ρ values consistently at or below ρ = -0.6, particularly at high probability of activation and/or low-to-moderate probability of degradation, leading us to conclude that the addition of macroautophagic degradation leads to a better simulation of biological reality. Furthermore, our experimental data demonstrated ρ values between -0.7 and -0.6 at both 20- and 40-min post-activation (**Fig 1D**), which suggests that a transient equilibrium is achieved between filament formation and degradation over a period of time leading to this steady negative correlation between puncta number and filament length. Interestingly, our simulations appear to replicate this stable equilibrium period over time before ρ reaches a state of steady increase due to a lack of remaining filaments and a dwindling supply of Bcl10 monomers.

Next, we sought to determine the effects of each individual probability parameter (p_attach,_ p_activate,_ and p_degrade_), as well as their interplay, on the concentration of filamentous Bcl10. As in **Fig 4**, for each set of probability parameters, we simulated 100 cells with random initial conditions and repeated each simulation 10 times (**Fig 5C**). As expected, increasing values of p_degrade_ in each parameter space led to a lower concentration of filamentous Bcl10 over all iterations of the simulation. Interestingly, both an increase in the rate of nucleation-site generation (represented by a greater p_activate_) and a lower activation barrier (represented by a greater p_attach_) led to a faster rate of decline in filamentous Bcl10 after the peak filament concentration was achieved. This result makes intuitive sense, as a greater number of activation sites would lead to a greater number of filaments, and thus a greater number of sites where degradation can occur; similarly, a lower activation barrier would lead to the initiation of a greater number of filaments earlier in the simulation, resulting in a more rapid increase in aggregate filament surface area for interaction with autophagosomes. When combined, a lower activation barrier and a greater activation of nucleation sites created a dynamic in which slight variations in p_degrade_ had a large effect on the rate of degradation of filamentous Bcl10 (**Fig 5C, bottom right**). In addition, this parameter configuration, when combined with moderate to high probability of degradation, leads to a situation where the concentration of Bcl10 filaments more rapidly approached 0, which better reflects the observed biological kinetic in which Bcl10 filaments reach their peak concentration between 20–30 min post-activation and are completely degraded by 60–90 min post-activation [13,15]. Furthermore, over the same intervals where filaments rapidly degrade, both the number of punctate Bcl10 structures and the number of active degradation sites remain constant or decrease, especially for larger values of p_degrade_ (**S2** and **S3** Figs). This aspect of the simulation is in contrast to real cell data (**Fig 3C**), suggesting that these simulations fail to recapitulate what is likely to be a more complex biological mechanism governing degradation of puncta.

We further explored how each of the three parameters in our simulation affected the variability of the response across the heterogeneous initial population of cells. For each of the 100 cells, we calculated the average number of iterations for the filament-length distribution to reach its maximum average size, n_max_, and compared this to the average number of iterations required for the average size to be half of the max, n_half_ (**Fig 5D**), for each of the twelve parameter configurations. Increasing the value of p_attach_, p_activate_, or p_degrade_ independently of the others led to decreased variability in outcomes, with an increase in p_activate_ having the greatest effect on variability in the response to an activation signal (i.e., heterogeneity in the time to peak response), and an increase in p_attach_ emphasizing changes in p_degrade_. Given our previous work demonstrating that TCR activation of NF-κB is digital [34], it is likely that decreased variability in response to a given amount of signal is desirable in biological reality.

In summary, we have developed a refined model of a critical T cell receptor signaling process that quantitatively captured the growth and decay dynamics of Bcl10 filaments and their heterogeneous length distribution. We determined that a high activation potential of nucleation sites can, when combined with a low Bcl10-nucleation barrier, provide the most predictable and homogenous response to T cell activation. Furthermore, we found that these same parameters (high activation potential of nucleation sites combined with a low nucleation barrier) results in the largest effect of degradation of Bcl10 filaments on the percentage of Bcl10 in filamentous form. These conditions ultimately allow the response dynamic to be maximally tunable via changes in the degradation rate. With regards to the biological governance of this signal transmission system, these data offer a mechanistic explanation for why Bcl10 polymerization is opposed by contemporaneous disassembly, via a mechanism that includes autophagic degradation of Bcl10 polymers.

## Discussion

In this work, we explored the mechanism and dynamics of Bcl10 filament degradation, a process that is a key regulatory element in the T cell receptor-to-NF-κB signal transduction pathway. Through computational analysis of super-resolution images, we demonstrated that Bcl10 filaments are shorter in length at 40 min post-TCR engagement than at 20 min, with an increase in punctate Bcl10 and a decrease in filaments longer than 1 μm. We demonstrated that autophagosomes were preferentially in contact with Bcl10 filaments, that these contacts increased over time, that at both 20 min and 40 min post-TCR engagement there was a preference for the contacts to occur near filament ends, and that by 40 min post-TCR engagement, autophagosomes colocalize with Bcl10 puncta in a non-random, significant manner. Together, these findings, along with our previous biochemical studies [18], imply that autophagosomes drive a degradative process that progressively shortens Bcl10 filaments, leaving behind remnants visualized as puncta which undergo slower degradation. Furthermore, we developed a stochastic Monte Carlo simulation of nucleation-limited filament growth and degradation, which we utilized to probe aspects of regulatory control of filamentous signal transduction bodies. Using this model, we ascertained that the rate of degradation was the most important element controlling the magnitude and dynamics of the response function, particularly when the homogeneity of the response has been maximized via a high activation potential and a low nucleation barrier.

Importantly, these results shed light on possible mechanisms of autophagic degradation in the poorly understood scenario in which the structures to be degraded are larger than the autophagosomes performing the degradation. We found that increased contact between autophagosomes and Bcl10 filaments corresponded to a progressive shortening of Bcl10 filament length, implying that autophagosomes degrade these large structures through a piecewise process rather than the currently understood model of enclosure of the entire volume of a given cargo. Further, we determined that filaments to which autophagosomes attached were more likely to experience attachment near filament ends, and that degradation models that target the ends of filaments rather than the mid-region best fit the experimental observations. However, we were unable to establish whether this autophagy process proceeds via end-based disassembly at the molecular scale, or via scission of larger regions near the end. The novel observation that the number of punctate structures in contact with autophagosomes increases significantly at 40 min vs 20 min could not be recapitulated in our model (**S2 and S3 Figs**), suggesting that the final degradation of Bcl10 filaments once they reach lengths <150nm and thus appear as puncta is inhibited or occurs by an independent mechanism. In addition, we note that our simulations are unable to account for the possible effects of proteosomal degradation on Bcl10 filament length. We have previously demonstrated that proteasomes are involved in the degradation of Bcl10 [18]; however, unpublished imaging work in our lab has not uncovered clustering of proteosomes around Bcl10 filaments, and thus their role in the degradation of oligomerized Bcl10 is unclear and cannot be reliably modeled.

The analysis methods that we developed for this study have broad applicability to computational image analysis and the study of dynamic signaling processes. To enhance the precision of our measurements of Bcl10-filament skeletons, we use the topological constraint that Bcl10 and CBM-complex macrostructures cannot branch. However, due to the proximity of Bcl10 filaments and the optical resolution of iSIM, we observed many apparent high-degree junctions made up of intersecting and seemingly connected filaments. Applying the assumption that these filaments are stiff, we developed a minimum-bending-energy scheme to parse and untangle nodes in the skeleton. The technique provides a simple and robust framework to predict the connected components on opposite sides of a high-degree junction while neglecting erroneous connections formed during medial-axis skeletonization. Furthermore, our image-based bootstrap-like method of random rearrangement of segmented objects has, to our knowledge, not been previously published, and could prove to be widely applicable in assessing patterns and colocalizations between structures in images. Radial-distribution functions are typically used to measure and statistically assess contacts and/or colocalizations between structures. However, such an analysis was not suitable in this manuscript because interactions between Bcl10 filaments and autophagosomes solely involve close-range contact with significant volumetric overlap. Filamentous signaling structures, particularly those whose core constituent protein contains a death domain superfamily element (as Bcl10 does), are a common motif in immunological signal transduction pathways [38–40], and many are downregulated via macroautophagy [40–43]. Thus, our model of nucleation-limited filament growth and degradation, with its multiple tunable parameters that can be used to fit the model to observed imaging data, may be applicable to a variety of autophagy-regulated signal transduction pathways and may furthermore reveal common mechanistic principles of immune regulation.

The simulations we developed offer intriguing insight into the regulatory control of T cell activation states. Higher values of p_activate_ and p_attach_ in the model led to increased heterogeneity in filament lengths; since our experimental observations include heterogeneous filament lengths, it is implied that the probability of activation of Carma1 in response to TCR engagement is likewise high in biological reality. Furthermore, these higher values of p_activate_ and p_attach_ in the model led to increased responsiveness to variations in degradation speed, along with decreased variability of the response from a variety of initial states, both outcomes that are beneficial to the overall control of T cell activation. Thus, it is probable that in activated T cells, Carma1 in the vicinity of the immunological synapse has a high likelihood of promoting Bcl10 filament nucleation, and further regulatory control of the activation potential of Carma1 would provide little overall benefit. Interestingly, based on the results of the simulations, governing the rate of filament disassembly (involving regulation of degradation via autophagy) has the greatest effect on the response dynamic. Moreover, the addition of the autophagy parameter to the simulations produced output data most similar to real cell data under conditions of high p_activate_ and p_attach_, further reinforcing the likely biological reality of high values for p_activate_ and p_attach_, as well as a key role for autophagy in governing the observed overall signaling kinetic in the TCR-to-NF-κB pathway. These conclusions provide support for the “mouse trap” hypothesis of nucleation-limited immune signaling, which proposes that pathway components are maintained in a supersaturated, easily activatable state until the required activation signal triggers a rapid response via polymerization that can best be downregulated by complete destruction of the component parts via autophagy [44]. Notably, autophagy has many regulatory control points and processes feedback from a wide variety of signaling pathways [45], providing myriad opportunities for crosstalk among T cell signaling inputs. In fact, given that there exists clear evidence that TCR engagement leads to a rapid increase in autophagosome formation [46,47], this process could be characterized as an incoherent feed-forward loop, with TCR signaling serving to both promote T cell activation and downregulate that same activation.

The results of our simulations, taken together, indicate that POLKADOTS filament growth and degradation exhibit the characteristics of an excitable system. Excitability is a phenomenon by which a system can undergo dramatic changes in behavior in response to small perturbations; components of such a system typically include non-linear thresholded/digital activation that initiates a fast, amplifying, positive feedback loop along with a slower, delayed, response-limiting negative feedback loop [48]. Excitable systems characteristics are observed in a variety of biological processes, including in the polymerization and depolymerization of cytoskeletal proteins during migration [49] and in ion channels during neuronal firing cascades [50]. Further, excitability is often observed when signaling networks reach a critical, near-phase-transition state [51,52], which induces dramatic changes to system dynamics, including the oligomerization of protein complexes via phase separation [53]. We have previously reported that the Bcl10-dependent TCR-to-NF-κB signaling pathway is characterized by digital activation, with increased stimulus beyond a certain threshold having little effect on the signaling response of individual T cells [34]. Thus, the predictions of behavior of POLKADOTS filament growth and degradation resulting from these simulations are consistent with our previous observations of the biological behavior of these signaling elements in living T cells.

Our analysis of Bcl10 filament formation and degradation suggest that this self-limiting all-or-nothing response is enabled by nucleation-limited filament assembly with a high probability of Carma1 activation in response to even minimal TCR engagement and a low nucleation barrier. The system is self-limiting, likely via autophagy operating on filament ends, which extends the lifetime of filaments and thus signal activation (a result not inconsistent with autophagy limiting the intensity of the signal by limiting the amount of Bcl10 in POLKADOTS filaments [18]). Disassembly via autophagy would result in a long refractory period due to protein degradation. More broadly, our results, when considered in combination with existing structural data [35], suggest that spatial organization of signaling components, specifically filament assembly and disassembly, may be widely used in biological signaling networks to enable robust all-or-nothing (i.e., digital) responses and/or self-limiting dynamics.

## Methods

### T cell stimulation and imaging

D10.G4.1 T cells were transduced with retroviruses expressing Bcl10-GFP and TagRFP-T-LC3 (referred to as RFP-LC3) as previously described [54]. Following antibiotic selection, cells were subcloned to ensure uniform imaging characteristics. The resultant D10 cell line was activated via plating on coverslips coated with anti-CD3 antibody (clone 145-2C11, BioXCell). Cells were fixed with 3% paraformaldehyde after the indicated incubation times and mounted for imaging on a custom-built instant structured illumination microscope (iSIM) utilizing a 1.45 NA oil immersion objective [36]. Images were collected as z-stacks inclusive of the entire volume of the cells with a 200 nm distance between z planes. Following collection, images were deconvolved using the Richardson-Lucy algorithm as previously described [36] and converted to a new isotropically-distributed 3D coordinate grid via 3D spline interpolation.

### Segmentation of Bcl10 and LC3

Fluorescent iSIM images of Bcl10 and LC3 were binarized using an intensity threshold on a cell-by-cell and channel-by-channel basis due to fluctuations in expression and intensity. Each binarized image was convolved with a 3x3x3 kernel with values 1/3^3^ and again binarized with a threshold of (2/3)^3^ so that pixels with at least 8 occupied nearest neighbor locations were considered. All small binary pixel-noise was removed. Colocalizations between Bcl10 and LC3 were determined by identifying the voxels which directly overlap in the two binary channels.

### Skeletonization and skeleton measurements of Bcl10

Each of the structures in the binary Bcl10 images were individually skeletonized using a MATLAB implementation of medial-axis thinning skeletonization [55–57]. Bcl10 skeletons sometimes contained erroneous spurs due to fluctuations in the volume from sensitivity to the intensity threshold and thus, filament skeletons were processed by iteratively removing spurs less than 6 voxels (150 nm) in end-to-end length, the approximate optical resolution of the iSIM. A representative example is demonstrated in **S1 Movie**. Structurally isolated skeletons less than 6 pixels (150 nm) in length were labeled as punctate as indicated in **Fig 1**.

Where multiple Bcl10 filaments overlapped, skeletons developed high-degree nodes where multiple filaments branches converged or intersected at a single branch point. This understanding of Bcl10 filament structure is inconsistent with current perspectives [14] and was treated as an imaging artifact. To estimate the appropriate skeleton segmentation in these regions, we instituted a minimum-bending-energy scheme under the assumption that filaments are less likely to develop large curvature, and thus chose the configuration that resulted in the smallest net bending energy. Bending energy for a simple elastic beam is proportional to 1/R^2^ where R is the radius of curvature of the bent region. Thus, we fit an 11-pixel segment centered at the high-degree junction between multiple skeletons to the surface of a sphere to calculate R for the segment.

Measurements from the ends of skeletons were calculated using the Floyd-Warshall algorithm where the reference point was the nearest degree-one node. Colocalizations between Bcl10 and LC3 were assigned the end-distance value from the nearest skeleton points in all overlapping voxels.

### Random rearrangement and resampling of segmented structures for statistical assessment

Bootstrapping is a statistical resampling procedure that allows for the estimate of a sampling distribution. Since resampling of a single fixed T cell at 20 min or 40 min post-activation is impossible, we developed an image-based resampling method to assess the sample distributions for the number and location of Bcl10-autophagosome contacts if the underlying biological processes are driven by randomness.

To carry out the bootstrapping procedure in this work, we identified three relevant cell structures from the 3-D super-resolution iSIM image: the cell boundary, binary Bcl10, and binary autophagosomes. For the resampling, we kept the location of the cell boundary and Bcl10 fixed. Then, we removed all autophagosomes from the image and separately saved each individual 3-D structure separately. Each 3-D autophagosome was then rotated by three randomly generated angles between 0 degrees and 360 degrees about the x-, y-, and z-axis. Finally, each randomly rotated autophagosome was placed one at a time in a random location within the cell boundary. If any voxel in the currently selected autophagosome fell outside of the boundary or overlapped with an existing autophagosome, then a new location was chosen. The procedure was repeated until all autophagosomes were placed. Upon completion, the contact calculations were repeated, including the number of Bcl10-autophagosome contacts per cell, and the location of each of the contacts along connected Bcl10-filament skeletons.

### Simulations of Bcl10 filament growth and degradation

Simulated cells were first initialized with a random number of Carma1 nucleation sites. It has been previously shown that Carma1 becomes activated at the immunological synapse and oligomerizes to nucleate Bcl10 filaments [20]. Here, we simplified this mechanism in our simulations by generating a fixed number of Carma1 oligomers that must become activated, and that then can nucleate one Bcl10 filament. The number of potential nucleation sites in the simulation were based on measurements from our experimental data (**Fig 4A**). Since the coefficients of variation of these data are large (0.75 and 0.59 for 20 min and 40 min, respectively), a normal distribution is not suitable to generate random numbers of Carma1 sites. Thus, in the simulations, we generated Carma1 based on a gamma distribution, and used the maximum-likelihood estimate of the parameters for the 20 min data of ∝ = 1.59 and β = 15.92. Furthermore, to enforce that Carma1 must be activated before nucleating Bcl10 filaments, we added an additional requirement that Carma1 must contact, and be activated by, the immunological synapse. To estimate the size of the immunological synapse, we used our image data to measure the proportion of the cell surface that is in contact with the anti-CD3-coated plate (**Fig 4A**). We found that the cell-surface contact area was best estimated by a normal distribution with parameters μ = 0.4 and σ^2^ = 0.025^2^.

To determine the range of monomeric Bcl10 present in cells, we performed quantitative western blots (**Fig 4B**). We determined that cells overexpressing Bcl10-GFP had approximately 540,000 ± 100,000 Bcl10 molecules per cell, and that these cells have approximately 30× the amount of endogenous Bcl10 found in wild-type D10 cells. We chose to simulate cells with a wild-type level of Bcl10 monomers, both to shorten the length of time and computing power needed to run the simulations and to reflect biological reality more accurately. Thus, simulated cells were initialized with a randomly generated number of Bcl10 monomers drawn from a normal distribution with parameters μ = 108,000 and σ^2^ = 3600^2^.

Lastly, for the simulations shown in **Fig 5**, a random number of autophagosomes were added to the simulated cells. Going back to the image data, we found that the coefficients of variation for the number of autophagosomes were large (0.57 and 0.40 for the 20 min and 40 min images, respectively, **Fig 4A**). We again fit the distribution of the number of autophagosomes to a gamma distribution and determined that the maximum-likelihood estimates of the joint distribution of 20-min and 40-min data to be ∝ = 4.30 and β = 7.90. Using these parameters, we sampled a gamma distribution to generate a random number of autophagosomes with which to initialize each cell.

The simulated evolution of filament growth and degradation was controlled by the activation of Carma1 at the immunological synapse, the nucleation of Bcl10 filaments by an active Carma1, the attachment of monomeric Bcl10 to nucleated Bcl10 filaments, the attachment of autophagosomes to Bcl10 filaments, and the degradation of Bcl10 filaments by autophagosomes. Each step in the evolution was mediated by both fixed and variable probabilities.

1. Activation of Carma1 by the engaged TCR.
  - Probability that Carma1 will collide with the membrane, p_collide_ = 0.1.
  - Probability that the membrane was part of the immunological synapse, p_synapse_ = N(μ = 0.4, σ^2^ = 0.025^2^).
  - Probability of Carma1 activation by the immunological synapse, p_activate_ (variable).
2. Growth of Bcl10 on activated Carma1.
  - Probability that Bcl10 will collide with activated Carma1 (per site), p_collide_ = 0.01.
  - Probability of attachment before the nucleation barrier has been reached, p_attach_ (variable).
  - Size of the nucleation barrier (i.e., the number of attachments before the attachment probability switches from p_attach_ to p_grow_), n_barrier_ = 50.
  - Probability of attachment after the nucleation barrier has been reached, p_grow_ = 0.4.
3. The degradation of Bcl10 by an attached autophagosome.
  - Probability of autophagosomes colliding with a Bcl10 filament, p_collide_ = 0.1.
  - Probability of autophagosomes attaching to a Bcl10 filament, p_attach_ = 0.1.
  - Probability of autophagosomes degrading attached-to filaments, p_degrade_ (variable).
    If autophagosomes attach to the growing filament end, degradation results in Bcl10 monomers being removed from the end;
    If autophagosomes attach to filament middle, degradation results in scission of the filament to two daughter filaments:
      1. Daughter filament 1, which remains associated with the nucleation site, is stable, does not undergo further growth, and can be further degraded by autophagosomes.
      2. Daughter filament 2 remains associated with the original autophagosome and continues to be degraded over subsequent iterations governed by the probability p_degrade_.
  - If attached autophagosomes do not degrade, the probability that they fall off the attached-to filament, p_detach_ = 0.1.

We used physical properties of Carma1 and Bcl10 to inform these interaction probabilities in the simulations. Bcl10 is 33 kDa and its cytosolic diffusion constant is on the order of D_Bcl10_ ≈ 10 μm^2^/s, whereas Carma1 is 130 kDa and thus has a cytosolic diffusion constant on the order of D_Carma1_ ≈ 10 μm^2^/s [39]. Via diffusion, the time it takes to traverse some distance d is t = d^2^/6D. T-cell diameters are on the order of d_T cell_ ≈ 10 μm, and thus the time it takes for Bcl10 and Carma1 molecules to traverse the cytosol are approximately 0.2 sec and 2 sec, respectively. Thus, the collision probability for Bcl10 was chosen to be 10× larger than that of Carma1. The other rate constants, such as p_grow_ and p_attach_, have not been determined experimentally, to our knowledge.

Simulations from **Fig 4** were performed without autophagosomes and terminated upon monomeric Bcl10 being completely converted to filamentous Bcl10. Simulations from **Fig 5** were performed for either 10,000 iterations or when the number of monomeric and filamentous Bcl10 reached zero, whichever came first.

## Supporting information

S1 Movie Semiautomatic segmentation, skeletonization, and pruning of Bcl10 filaments. Representative segmented Bcl10 filament superimposed medial-axis skeletons. (Left) Raw medial-axis skeleton. The skeleton contains several small spurs which occur because of voxel noise on the surface of the filament. (Middle) Medial-axis skeleton after moderate pruning. Some spurs are removed. (Right) Medial-axis skeleton after full pruning. All spurs are removed and only the main-body skeleton remains.
(MP4)

S1 Table Statistics for Fig 1. (XLSX)

S2 Table Statistics for Fig 2C. (XLSX)

S3 Table Statistics for Fig 3. (XLSX)

S4 Table Statistics for Fig 4E. Average filament lengths, filament length variances, and rho correlation values for simulations in Fig 4E. Numbers highlighted in red most closely match observed values.
(XLSX)

S1 Fig Shape statistics of autophagosomes 20 min and 40 min post-activation. (A) Distribution of autophagosome 3-D volumes. (B) Distribution of autophagosome 3-D surface areas. (C) Distribution of autophagosome sphericities. Sphericity of one is a perfect sphere and sphericity of zero is a flat disk.
(TIF)

S2 Fig Average number of Bcl10 puncta in simulations over time. Average number of Bcl10 puncta over time in selected simulation configurations with low, moderate, and high values of p_degrade_. The black outlined graphs correspond to the configurations shown in **Fig 5**.
(EPS)

S3 Fig Average number of active degradation sites in simulations over time. Average number of autophagosome simulation sites over time in selected simulation configurations with low, moderate, and high values of p_degrade_. The black outlined graphs correspond to the configurations shown in **Fig 5**.
(EPS)
