# Supplementary figures and images for: Signaling through polymerization and degradation: Analysis and simulations of T cell activation mediated by Bcl10
Source: PLoS Comput Biol. 2021 May 20;17(5):e1007986. doi: 10.1371/journal.pcbi.1007986 (PMC8184007; doi:10.1371/journal.pcbi.1007986)

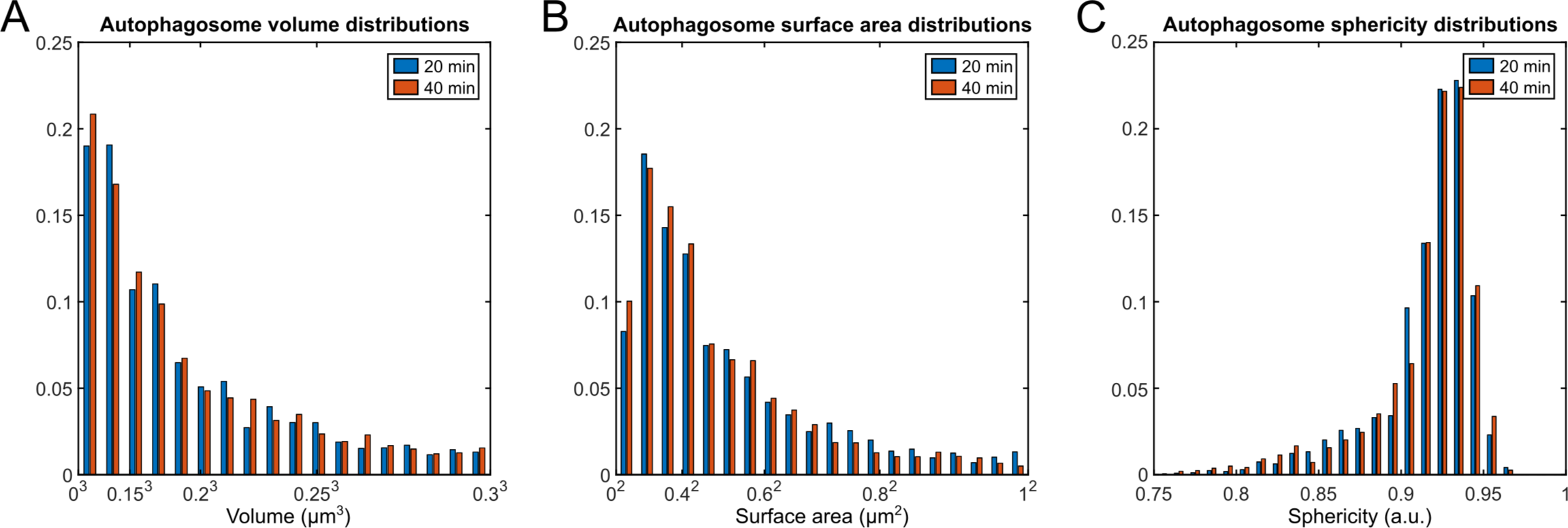

Supplement: S1 Fig — (A) Distribution of autophagosome 3-D volumes. (B) Distribution of autophagosome 3-D surface areas. (C) Distribution of autophagosome sphericities. Sphericity of one is a perfect sphere and sphericity of zero is a flat disk. (TIF) [file pcbi.1007986.s006.tif]
